# Eliminating Cu–Cu Bonding Interfaces Using Electroplated Copper and (111)-Oriented Nanotwinned Copper

**DOI:** 10.3390/ma17143467

**Published:** 2024-07-13

**Authors:** Tsan-Feng Lu, Yuan-Fu Cheng, Pei-Wen Wang, Yu-Ting Yen, YewChung Sermon Wu

**Affiliations:** Department of Materials Science and Engineering, National Yang Ming Chiao Tung University, Hsinchu 30010, Taiwan; s0881513.c@nycu.edu.tw (T.-F.L.); weddie6231.11@nycu.edu.tw (Y.-F.C.); nycu3116.en11@nycu.edu.tw (P.-W.W.); yu.en11@nycu.edu.tw (Y.-T.Y.)

**Keywords:** grain size distribution, grain boundary energy, Cu–Cu direct bonding, microstructure, grains and interfaces, nanotwinned Cu

## Abstract

Cu–Cu joints have been adopted for ultra-high-density packaging for high-end devices. However, the atomic diffusion rate is notably low at the preferred processing temperature, resulting in clear and distinct weak bonding interfaces, which, in turn, lead to reliability issues. In this study, a new method for eliminating the bonding interfaces using two types of Cu films in Cu–Cu bonding is proposed. The difference in grain size was utilized as the primary driving force for the migration of bonding interfaces/interfacial grain boundaries. Additionally, the columnar nanotwinned Cu structure acted as a secondary driving force, making the migration more significant. When bonded at 300 °C, the grains from one side grew and extended to the bottom, eliminating the bonding interfaces. A mechanism for the evolution of the Cu bonding interfaces/interfacial grain boundaries is proposed.

## 1. Introduction

The advancement of integrated circuit technology is primarily driven by four key metrics: power consumption, performance, area, and cost. However, as Moore’s Law approaches its limitations, three-dimensional integrated circuits (3D ICs) are considered one of the most practical solutions. They offer benefits such as heterogeneous integration, reduced form factors, lower power consumption, decreased RC delay, and reduced costs through shorter interconnects and vertical stacking [1,2,3]. Currently, Cu stands as one of the most promising materials for interconnects due to its excellent electrical conductivity and resistance to electromigration [4,5,6,7]. Additionally, Cu–Cu bonding technology meets the increasingly high demands for input/output (I/O) density and reliability, which pose challenges for traditional solder interconnects. However, Cu–Cu bonding technology also has its own reliability issues, which have been extensively studied recently [8,9].

The mechanism of conventional thermocompression bonding involves the interdiffusion of atoms and grain growth at the bonding interfaces, which can eliminate bonding interfaces at high temperatures. However, bonding temperatures below 300 °C are preferred, as elevated temperatures may lead to the warping of silicon wafers due to the mismatch in thermal expansion coefficients among Si, Cu, and dielectric materials, resulting in reliability issues [10,11,12]. Therefore, accelerating Cu diffusion is necessary for low-temperature bonding.

Chang et al. [13] demonstrated the elimination of bonding interfaces by utilizing nanotwinned Cu, which underwent abnormal grain growth (AGG) at 250 °C and formed single crystals, effectively eliminating the bonding interfaces. As a result, the bonding strength of Cu joints increased from 46.1 MPa to 57.1 MPa after the bonding interfaces were eliminated. In our previous study, we employed a surface modification technique using epoxy to create a dual-layer microstructure [14]. This approach aimed to eliminate bonding interfaces through AGG of the fine-grained layer. Additionally, in another experiment, we promoted grain growth and eliminated Cu–Cu bonding interfaces by applying a surface quenching treatment [15].

In this study, we propose a novel and simple method to promote grain growth and eliminate bonding interfaces. By employing different electroplating processes, two types of Cu films with varying grain sizes and microstructures were produced. This method does not require additional processing equipment and is compatible with semiconductor manufacturing processes. In this study, the properties of grain boundary energy differences were utilized to form higher-quality bonding interfaces at lower temperatures.

## 2. Experiment

### 2.1. Cu Film Electrodeposition

In this study, two types of electroplated Cu films on Si substrates were used. One was a regular electroplated Cu (referred to as BCu) film and the other was a highly (111)-oriented nanotwinned Cu (referred to as NtCu) film. The substrate of the BCu film consisted of a 70 nm thick SiO_2_ layer, a 20 nm thick Ta layer, and a 600 nm thick Cu seed layer. Subsequently, a 1.4 μm thick Cu film was electroplated onto the substrate.

The highly (111)-oriented NtCu films were fabricated using a direct current (DC) electroplating process. This method, discovered by Tao-Chi Liu et al. [16], has been extensively studied. The electrolyte solution was composed of high-purity CuSO_4_ with 0.8 M Cu cations, and a high-purity (99.99%) copper sheet served as the cathode. To promote the formation of nanotwins, surfactants provided by Chemleaders Inc. (Hsinchu, Taiwan) were introduced into the electrolyte, alongside 40 mg/L of HCl. During the electroplating process, the electrolyte was thoroughly mixed with a magnetic stirrer operating at 1200 rpm. Additionally, a DC current density of 1200 A/m^2^ was maintained throughout. After the electrodeposition process, the NtCu films underwent planarization through chemical mechanical polishing (CMP). This process is consistent with previous studies [14].

### 2.2. Sample Pretreatment

After the CMP process, all samples were diced into 1 × 1 cm^2^ pieces for subsequent cleaning and bonding processes. The pieces were ultrasonically cleaned with acetone and then cleaned with a citric acid solution, rinsed with acetone and deionized (DI) water, and, finally, purged with N_2_ gas.

### 2.3. Bonding Process

After the pretreatment, three types of samples were placed in a differential thermal expansion fixture made of aluminum and stainless steel for the bonding process: (a) B/B (BCu-to-BCu bonding sample), (b) Nt/Nt (NtCu-to-NtCu bonding sample), and (c) B/Nt (BCu-to-NtCu bonding sample). This fixture was identical to the one employed in our prior research [14]. The bonding process was conducted at 300 °C for 1 h and 2 h, respectively, under ordinary vacuum conditions (1.33 × 10^−1^ Pa). With the increase in bonding temperature, the compressive stress on the sample stack also increased due to the different thermal expansion rates of the fixture’s materials. At 300 °C, the compressive stress was calculated to be 65.56 MPa. However, it was challenging to accurately measure the actual stress because the Cu films underwent plastic deformation/surface creep at elevated temperatures.

### 2.4. Analysis Methods

Prior to the bonding process, the surface roughness (RMS) of the Cu surfaces was measured over an area of 10 × 10 µm^2^ using an atomic force microscope (AFM, Bruker Dimension Icon Scanning Probe Microscope, Billerica, MA, USA).

Information on the structure and crystallographic orientation based on Kikuchi patterns was obtained using electron backscattered diffraction (EBSD) with an EBSD detector (Oxford Nordlys Max3, Abingdon-on-Thames, UK). This analysis utilized a scanning electron microscope (SEM, JEOL JSM-7800F, Tokyo, Japan) operating at 20 kV. OIM™ post-processing software (https://www.edax.com/products/ebsd/oim-analysis accessed on 9 June 2024, TexSEM Laboratories, Draper, UT, USA) was used to obtain average grain size and crystallographic texture data. This analysis was conducted via plan-view EBSD of the Cu film surfaces.

To evaluate the quality of the bonded interface, we utilized a dual-beam focused ion beam (DB-FIB, Helios NanoLab 650, FEI, Hillsboro, OR, USA) to investigate the microstructure at the bonding interfaces. The bonded samples were prepared by grinding and polishing their cross-sections. To achieve a clearer view of the interface, a light etching process using FIB was employed to clean both the surface and the bonding interface.

## 3. Results and Discussion

### 3.1. Surface Roughness and Crystallographic Information of Cu Films

**Figure 1:**
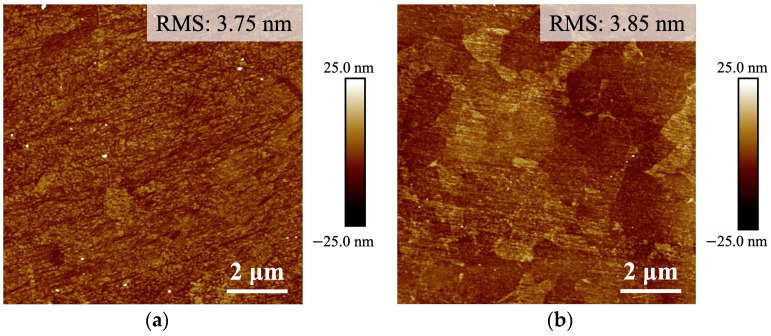
AFM topography images of (**a**) BCu and (**b**) NtCu films.

The AFM surface topographies of the Cu films, as depicted in Figure 1, revealed that the BCu film and NtCu film had surface roughness (RMS) values of 3.75 nm and 3.60 nm, respectively. The surface roughness of both Cu samples showed minimal differences after CMP. This helped to minimize the influence of surface roughness on the formation of interfacial voids.

**Figure 2:**
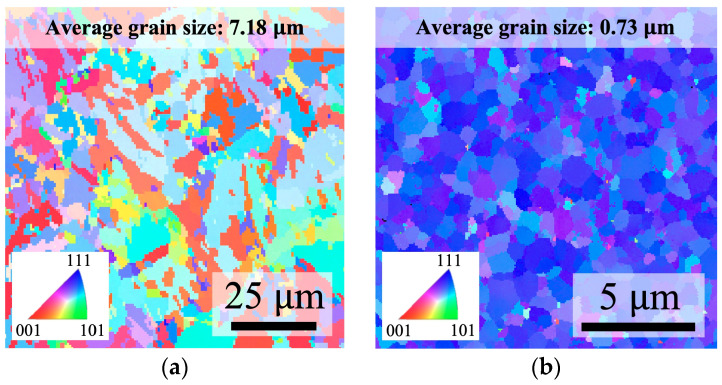
Plane-view EBSD OIM images of (**a**) BCu and (**b**) NtCu films. The BCu film exhibited random orientation, while the NtCu film was highly (111)-oriented. The average grain sizes of the BCu film and NtCu film were 7.18 μm and 0.73 μm, respectively.

The EBSD OIM images of the as-deposited BCu and NtCu surfaces are shown in Figure 2, where colors represent the crystalline orientation. The EBSD OIM image of the BCu surface displayed relatively larger grains and a random orientation, without any particular preferred orientation. In contrast, the grain size of the NtCu surface was much smaller and exhibited a highly (111)-preferred orientation. The average grain sizes of the BCu surface and the NtCu surface were measured to be 7.18 µm and 0.73 µm, respectively. This result confirmed the significant grain size difference between the two types of Cu films. Additionally, the highly (111)-preferred orientation on the NtCu surface was beneficial for the healing of the bonding interface as it had fast surface diffusion, which may have provided an opportunity for atoms to move along the bonding interfaces and fill the voids.

### 3.2. Effect of Grain Boundary Energy on Bonding Interfaces

**Figure 3:**
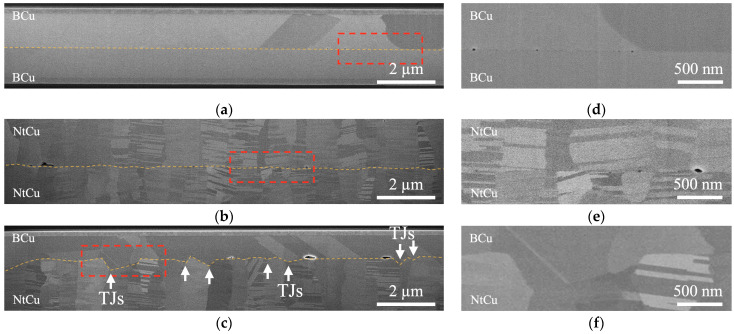
Cross-sectional SEM images after bonding at 300 °C for 1 h: (**a**) B/B, (**b**) Nt/Nt, and (**c**) B/Nt. (**d**) High-magnification image of the red dotted area in (**a**). (**e**) High-magnification image of the red dotted area in (**b**). (**f**) High-magnification image of the red dotted area in (**c**). Note: In (**a**–**c**), the bonding interfaces/IGBs are represented by orange dashed lines, and in (**c**), the white arrows point to the locations of triple junctions (TJs).

The extension of grain growth across the bonding interfaces was critical in determining the mechanical properties and reliability of the Cu joints. The cross-sectional SEM images shown in Figure 3 illustrate the bonding interfaces/interfacial grain boundaries (IGBs) observed after the samples were bonded at 300 °C for 1 h. As shown in Figure 3a,b,d,e, the B/B sample exhibited a clear and distinct bonding interface/IGBs. Similarly, the Nt/Nt sample, which featured columnar grains and nanotwinned structures, showed minimal grain growth across the bonding interface and remained intact. The flat plane of the bonding interface indicated a relatively weak bond [17,18,19], suggesting limited diffusion between the two Cu films.

However, as shown in Figure 3c,f, the B/Nt sample exhibited a zigzag shape, which could be attributed to a larger difference in grain size between the two types of Cu film. Due to the thermodynamic instability of fine-grained structures with high boundary energy, atoms tend to randomly migrate from high-energy small grains (NtCu) to low-energy large grains (BCu). This high grain boundary energy serves as a ‘macroscopic’ driving force for grain growth and Cu interdiffusion [20]. Most of the Cu grains on the BCu side grew into the NtCu side, consuming small grains and transforming into large grains. Therefore, the growth of interface grains eliminated the original bonding interface.

**Figure 4:**
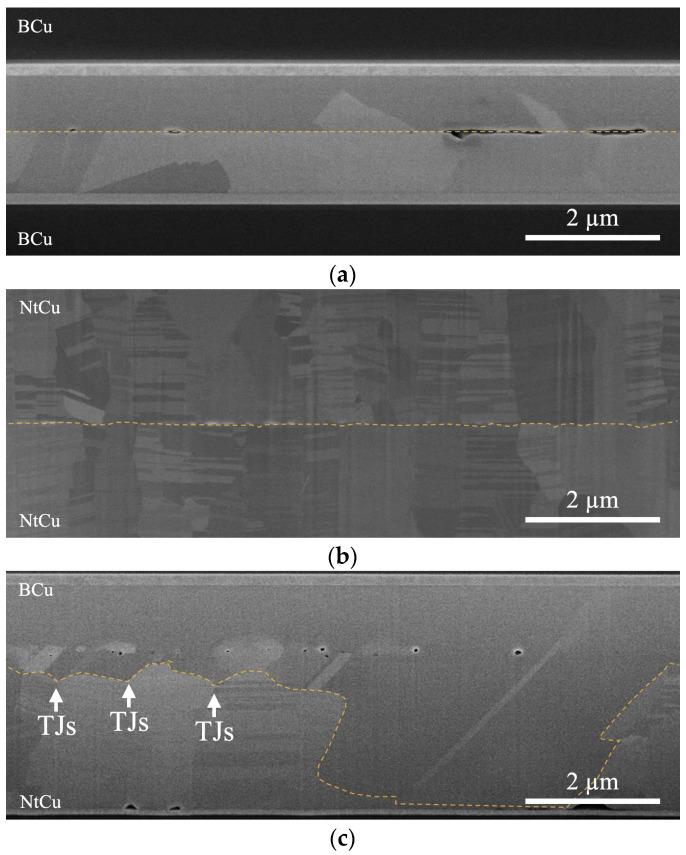
Cross-sectional SEM images after bonding at 300 °C for 2 h: (**a**) B/B, (**b**) Nt/Nt, and (**c**) B/Nt. Note: The bonding interfaces/IGBs are depicted with orange dashed lines and the white arrows in (**c**) indicate the locations of triple junctions (TJs).

To further understand the migration behavior of bonding interfaces/IGBs, we bonded the Cu films at 300 °C for 2 h, as shown in Figure 4. The B/B sample and the Nt/Nt sample remained consistent with the aforementioned results, with the bonding interfaces/IGBs staying flat or showing slight migration. Interestingly, for the B/Nt sample, most of the BCu-side grains grew into the NtCu side and extended to the bottom when the bonding time was increased to 2 h. The majority of the columnar grains with nanotwinned structures were consumed and transformed into large grains.

**Figure 5:**
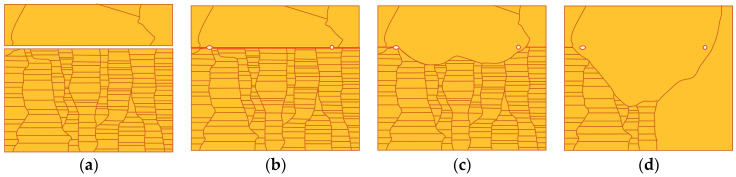
A schematic diagram illustrating the evolution of the B/Nt bonding interfaces/IGBs. (**a**) Cu films in contact at room temperature. (**b**) IGBs formed during the annealing process. (**c**) Grain growth across the IGBs consumed twin boundaries to reduce the grain boundary energy. (**d**) The grains on the BCu side further grew into the NtCu side and extended to the bottom.

To discuss the behavior of grain growth, Figure 5 shows the changes in the B/Nt bonding interfaces/IGBs. Figure 5a shows that voids appeared at the bonding interface when the Cu films were contacted at room temperature. This phenomenon was attributed to the surface roughness present on the Cu films. When the sample was subjected to thermal compression bonding, the Cu atoms diffused from the contact area to the void area to shrink the interfacial voids [17,19,21,22], and the contact area became IGBs, as shown by the red lines in Figure 5b. After the IGBs formed, grain growth on the BCu side initiated at the triple junctions (TJs) of the IGBs, as illustrated in Figure 5c. This transition was driven by the high energy associated with the “T”-type grain boundary junctions at the Cu–Cu bonding interfaces. In an effort to minimize this energy, the system strove to rearrange triple junctions to achieve a more uniform distribution of grain boundary angles [23]. This phenomenon was also observed in our previous study [14,15]. Additionally, changes in grain size distribution affected the kinetics of grain growth. A larger difference in grain size resulted in greater grain boundary curvature, which, in turn, created a greater driving force for boundaries to engulf smaller grains (see Ostwald ripening [24,25,26,27]). Consequently, grain growth occurred to diminish the significant grain boundary energy, provided there was sufficient thermal energy to facilitate grain boundary migration. With increasing annealing time, the grains on the BCu side further grew into the NtCu side and extended to the bottom, as shown in Figure 5d. Juang et al. [28] proposed that in (111)-oriented NtCu, characterized by columnar grains, each grain comprises parallel twin lamellae with a high density of coherent twin boundaries (CTBs). All columnar grains share a common tilt axis, resulting in all grain boundaries being tilt-type grain boundaries. Furthermore, tilt-type columnar grain boundaries (CGBs) contain a high density of TJs where CTBs intersect CGBs. These high-density TJs possess higher energy. Therefore, the grains on the BCu side grew downward from the TJs at the bonding interfaces, eliminating the high-density TJs, which helped reduce the system energy. Additionally, the elimination of the high density of CTBs also contributed to lowering the system energy, which is why we observed the grains growing slightly horizontally, as shown in Figure 4c.

## 4. Conclusions

In this study, we focused on the driving force of grain growth to eliminate the bonding interfaces. The primary driving force was the difference in grain size. The two types of microstructures in the Cu–Cu bonding, characterized by a larger difference in grain size, were analyzed and measured by EBSD. At 300 °C for 1 h, the B/Nt sample exhibited a significant zigzag-shaped bonding interface, while the B/B and Nt/Nt samples remained almost flat. At 300 °C for 2 h, the grains on the BCu side of the B/Nt sample further grew into the NtCu side and extended to the bottom. In addition to the difference in grain size serving as the primary driving force, the high density of twin boundaries in NtCu also provided an additional driving force, facilitating the migration of bonding interfaces/IGBs. This result holds promise for low-temperature bonding and the elimination of bonding interfaces, indicating potential for enhancement.

## Data Availability

The data supporting the findings of this study are available from the corresponding author upon reasonable request.
